# Seasonal recharge and mean residence times of soil and epikarst water in a small karst catchment of southwest China

**DOI:** 10.1038/srep10215

**Published:** 2015-05-11

**Authors:** Ke Hu, Hongsong Chen, Yunpeng Nie, Kelin Wang

**Affiliations:** 1 Key Laboratory of Agro-ecological Processes in Subtropical Region, Institute of Subtropical Agriculture, Chinese Academy of Sciences, Changsha, Hunan, 410125, China; 2 Huanjiang Observation and Research Station for Karst Ecosystems, Chinese Academy of Sciences, Huanjiang, Guangxi 547100, China; 3 Graduate University of Chinese Academy of Sciences, Beijing, 100049

## Abstract

Soil and epikarst play an important role in the hydrological cycle in karst regions. This paper focuses on investigating the seasonal recharge and mean residence time (MRT) of soil water and epikarst water in a small karst catchment of southwest China. The deuterium contents in precipitation, creek, soil baseflow (direct recharge of the saturated soil water to the stream), epikarst spring, and soil waters were monitored weekly for two years, and MRT was calculated by an exponential model (EM) and a dispersion model (DM). The obvious seasonal variation of deuterium in rainfall was buffered in epikarst water, indicating sufficient water mixing. Soil baseflow contained less rainy-season rainwater than epikarst spring discharge, reflecting the retarded effect of soil thickness on rainwater recharge. MRTs of all water bodies were 41-71 weeks, and soils in the depression extended those of shallow groundwater. This demonstrated that the deep soil layer played an important role in karst hydrological processes in the study catchment. The creek was recharged mostly by rainfall through epikarst, indicating its crucial role in water circulation. These results showed epikarst had a strong water-holding capacity and also delayed water contact time with dolomite.

Seasonal recharge and mean residence time (MRT) are important for a well-developed understanding of water movement and storage in subsurface systems^1^,^2^,^3^,^4^. Seasonal recharge reflects the relative importance of rainy-season versus dry-season precipitation and provides fundamental information to a catchment’s water balance at annual scales. MRT is the average time water spends in a subsurface system before it emerges as surface flow. Knowledge of MRT is essential for evaluating the connectivity of a subsurface water system and for examining a catchment’s response to land-use or environment changes^5^,^6^,^7^.

Karst landscapes are found in carbonate bedrock sites which exhibit highly heterogeneous hydrological processes due to uneven distribution of dissolving rock, and they are characterized by sinkholes, sinking streams, closed depressions, subterranean drainage and caves^8^,^9^,^10^,^11^,^12^. Circulation and storage of groundwater in karst aquifers are obviously different from those in non-karst areas. Pockets of saturated soils occur atop karst (epikarst), and cracks and fissures in bedrock result in karst conduits^13^,^14^. Epikarst is a perched saturated zone above the groundwater table, and it stores part of the infiltrated rainwater and controls the water circulation dynamics^3^,^15^. The shallow and discontinuous soils and fissured epikarst comprise a double-porosity media. This makes water percolation and movement complex and difficult to be deciphered in karst regions.

Traditional hydrogeological investigation tools used in porous environments are not appropriate to identify the role of the epikarst in the complicated karst aquifers^16^. This circumstance results from the fact that the traditional methods cannot be used to track water infiltration and percolation under conditions of complex flow processes in an unsaturated zone^17^,^18^. Recently, natural tracers, such as oxygen-18 and deuterium concentrations in seasonal dynamics of soil water, stream, and spring water at the catchment scale, have become the method of choice for investigating hydrological processes^4^,^19^,^20^,^21^. This method is based upon the distinct seasonal changes of deuterium and oxygen-18 concentrations presented in rainfall and traced in output water^22^,^23^,^24^. Information about seasonal recharge and MRT under different subsurface systems can be explained more easily by use of stable isotopic methods than by use of other physical techniques. The seasonal recharge can be evaluated by long-term stable isotopic methods. Moreover, deuterium and oxygen-18 concentrations are nearly only affected by hydrologic flow paths and evaporation. Consequently, the different average annual deuterium and oxygen-18 values between rainfall and subsurface water could be seen as the seasonal recharge bias^1^,^2^,^25^.

Besides seasonal recharge of groundwater, stable isotopes also can be used to reveal more complex hydrological issues. For example, these techniques have been used in recent research of MRT of groundwater to evaluate the storage and flux of subsurface water. The attenuation of precipitation signals by the study catchment has been used to evaluate the MRT for subsurface water using sinusoidal relationships between input and output waters and other lumped mathematical models that describe flow-path distributions^5^,^7^. Based on different flow paths, exponential model (EM) and dispersion model (DM) are applicable for unconfined and confined aquifers, respectively^26^. Previous studies have shown that, in the shallow soil area, DM approximates the MRT of tracers with high accuracy in fissured-porous aquifers, which are more similar to karst areas^5^,^27^,^28^. The exchange between immobile water in the micropores and mobile water in the fissures and fractures gives a steady-state condition to use DM^5^,^15^. Furthermore, the DM also can describe the realistic water exit time distribution^7^,^29^. Differently, EM is assumed to fit when there is no water exchange among different flow lines but well mixed in the outlet, and it has the exponential distribution of transit times^6^. If new water fraction has small effect on the output water resource, the EM perhaps has the good fit^6^,^7^. However, EM did not adapt for the soil water where there was frequent exchange between mobile and immobile water. Consequently, MRT calculated with EM might have a bias which did not meet the real flow patterns.

The hydrological functions of soil and epikarst probably have been underestimated because of poor understanding of hydrologic relationships to their structures and thickness^8^,^30^,^31^. Many previous studies had showed that catchment topography and aquifer structures controlled groundwater movement in sites with shallow soils^32^,^33^. Maloszewski *et al.*^5^ reported that a karstic aquifer was characterized by two interconnected parallel flow systems: (a) a fissured-porous aquifer, and (b) karstic channels in the limestone. Moreover, they distinguished the fast and slow flow systems correspondingly^5^. However, this situation might be different in the experimental site with dolomite. Relatively deep soil cover and poor connectivity of aquifers existed in dolomitic karst areas, because there were different dissolution patterns in carbonate rocks of these types^9^. On the one hand, Soulsby *et al.*^34^ claimed that the influence of catchment topography on MRT was largely mediated by its influence on soil cover and distribution in mountain catchment of Scotland. Moreover, the soil hydrology in a mountain forest catchment had a strong influence on MRT^33^. On the other hand, the longer vertical flow-path length for outlet water in mountainous regions might cause longer MRT relative to epikarst spring locations. This has been substantiated by McGuire *et al.*^6^ in that flow paths from unsaturated soil to groundwater are shorter than vertical recharge. Assano *et al.*^35^ also suggested that the length of vertical infiltration in an unsaturated zone controlled MRTs of soil water and transient groundwater. However, the influence of soil cover and thickness on MRT, seasonal recharge and water movement in epikarst was usually neglected in karst studies. Either only focusing on epikarst water movement or soil water movement would lead to mistakes. What’s more, epikarst contacts between rock and soil typically consist of an unconsolidated layer of rock mixed with soil^30^ and the hydrologic connection between footslopes and depression is continuous by means of lateral flow producing smaller responses to rainfall^36^. Consequently, the understanding of hydrological functions of both epikarst and the soil zone is urgent for knowing water movement in karst catchment.

The overall objectives of this paper are to evaluate the seasonal recharge and MRT of creek, soil baseflow (a flux of water going directly from the saturated soil to the stream), soil water and epikarst spring for better understanding the hydrological functions of soils and epikarst, based on seasonal variation of stable isotopes in a small karst catchment of southwest China. Moreover, the relationship among the above-mentioned water bodies is also discussed for investigating the influences of soil cover and thickness on water movement in underlying epikarst.

## Results

### Rainfall amount and deuterium content

**Figure 1:**
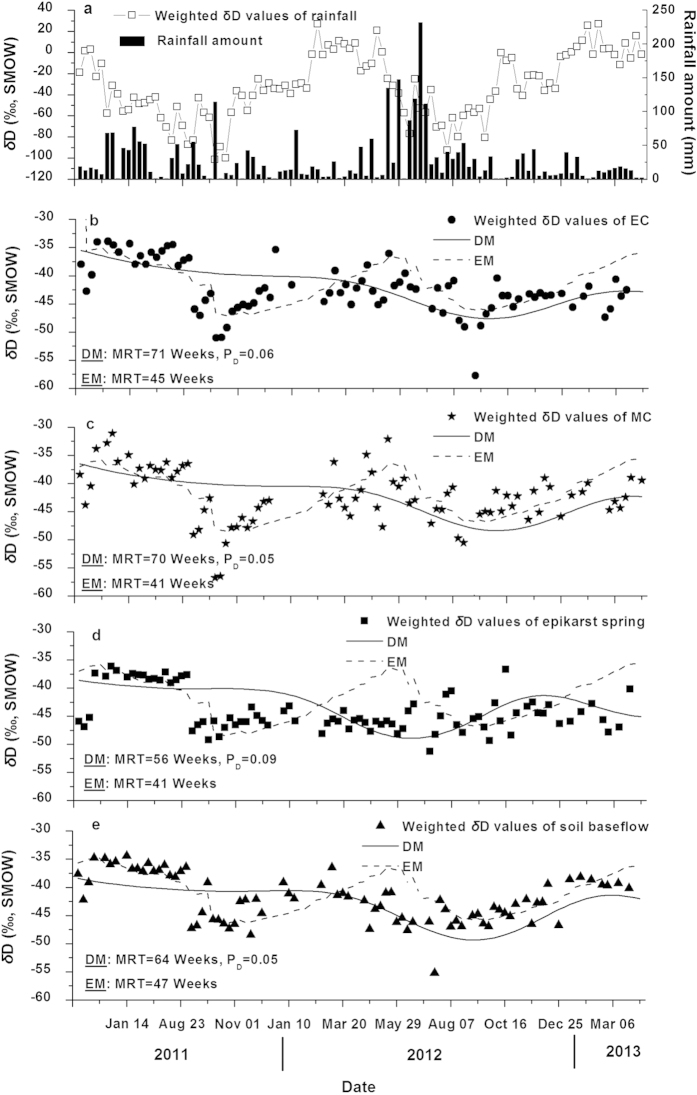
Seasonal variation of deuterium contents in rainfall (**a**), EC (**b**), MC (**c**), epikarst spring (**d**) and soil baseflow (**e**) and EM and DM fits in experimental site from April 10, 2011 to April 9, 2013.

The total rainfall from May to October covered by about 70% of those during the study period (Fig. 1a). The *δ*D (deuterium content) values in rainfall manifested strong seasonal variation and presented the characteristics of a sinusoidal wave with two sinusoidal cycles of 58‰ peak-to-peak variation during the sampling period. Although there was obvious seasonal variation for *δ*D over a year, no clear relationship between event-based precipitation amount and *δ*D was found. When rainfall amount was high, however, more depleted values of stable isotopes tended to be obtained.

Table 1 presents the number of weeks for different rainfall amounts during dry and rainy seasons from April 2011 to April 2013. The weeks with 0-10 mm rainfall were similar during dry and rainy seasons. However, the weeks with >50 mm rainfall during rainy season were more than those during dry season. This implied that rainy-season water had more opportunities to produce bypass flow in shallow soils and to recharge the deep soil layer as well as the underlying epikarst zone.

### Soil distribution

The distribution of soils was the premise for understanding water storage in soil zone. The results (Table 2) showed that the average soil depth and its standard deviation increased gradually with the increase of slope elevation. This indicated that the depression had deeper soil cover but higher uneven distribution than the slopes. The major soil texture was sandy soil in the top slopes, but clay loam soil in the depression and footslopes. The tendency of the above-mentioned soil distribution demonstrated that the water storage and hydrological function of soil at slopes were weaker than those in the depression. That is to say, the soil in the depression and footslopes, where soil thickness was deeper, played more important role in the water regulation relative to upslope soil zone.

### Water response of creek and epikarst spring to rainfall

**Figure 2:**
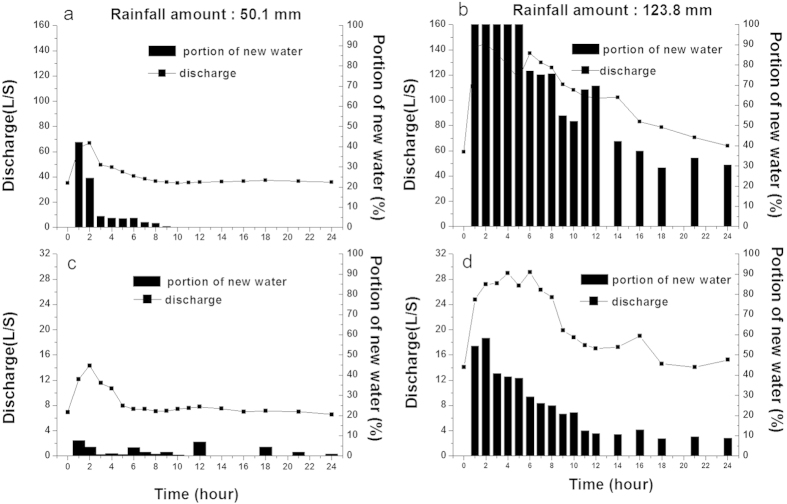
Water response of EC and epikarst spring to rainfall event on April 5 (**a**, **c**) and March 29 (**b**, **d**), 2012, respectively.

Water responses of the end of the creek (EC) and the epikarst spring to two typical rainfall events (rainfall amount were 50.1 and 123.8 mm, respectively) were different (Fig. 2). However, the changes of discharge between the EC and the epikarst spring were similar. The discharge increased rapidly with the first three hours and then decreased approximately 21 hours later. However, the portions of new water in the EC and the epikarst spring were different which were influenced by rainfall amount, and they were higher in the EC. During low rainfall events, the portions of new water in the EC and epikarst spring were all lower than 40% and 10%, respectively, even during the period with the highest discharge. However, during the high rainfall event, the portion of new water in the EC ranged from 25% to 100% and it was distinctly higher than that during the lowest rainfall event. Yet, epikarst spring also had lower than 30% new water most of the times. This is consistent with the supposition that the rainfall with high amounts tends to produce surface runoff, and fast flow channels are rare in the subsurface system of the study area.

### Seasonal recharges of different water bodies

In comparing with the change in rainfall isotopic content, the seasonal variations in deuterium contents of the middle of the creek (MC), EC, soil baseflow, and epikarst spring were sufficiently buffered as shown in Fig. 1. This indicated that each kind of water body sampled had small response to rainfall events, and the water was mixed sufficiently in the epikarst zone and deep soil zone.

Table 3 shows the average annual weighted *δ*D values in the MC, EC, soil baseflow, and epikarst spring were close to those in rainfall during the rainy season. This demonstrated that major recharge of these water bodies occurred during the rainy season. Similar weighted *δ*D values were found in the EC, MC and soil baseflow but they were more enriched than those in the epikarst spring. This suggested that the epikarst spring was recharged more by rainy-season rainwater than the other water bodies. Moreover, seasonal variation of *δ*D in rainfall was strongly buffered in the creek, soil baseflow, epikarst spring and deep soil layer. This indicated that epikarst and soils controlled the rainfall recharge dynamics. The average annual weighted *δ*D values of soil waters became depleted along with increase in soil depth. Deep soil waters (60-100 cm) in the depression had more enriched average weighted *δ*D values than those at footslopes.

The recharge portion in the EC, MC, soil baseflow and epikarst spring was lower than the contribution of dry-season rainfall. This indicated that the recharge rate of rainy-season rainfall was higher than that of dry-season rainfall. The recharge portion in the epikarst spring was lower than those in the EC, MC and soil baseflow. In addition, soil waters had decreased recharge portion with increase in depth. Their recharge portions varied from 34.56% to 63.49% at 20 and 40 cm depths, but were lower than 26.19% at 60, 80 and 100 cm depths. These revealed that a large number of rainy-season rainfall did not recharge the shallow soil layers (0-40 cm) but produced bypass flow.

The highest coefficient of variation (CV) of *δ*D (96.79%) was found in precipitation. The CV in *δ*D of soil water decreased with increase in depth from 91.22% to 8.07% but it was sharply decreased in the SD-1 at 60 to 100 cm depth. The EC, MC, soil baseflow and epikarst spring had similar small CVs to the SD-1 at 60-100 cm depths, which were much lower than the others. This indicated that water mixed well in deep soil in the depression and epikarst zone. However, CV of *δ*D in the SD-3 was special because of the existence of sandy soil, and fast infiltration might cause the less mixing.

### MRTs of different water bodies

The MRTs of water in the MC, EC, soil baseflow, epikarst spring and soil zone, which were calculated by DM and EM (except soil waters), are illustrated in Table 4 and Fig. 1. The relative lower SIGMAs were found in the EC, MC, soil baseflow and epikarst spring, and the higher SIGMAs were obtained in soil waters. Based on the fitting lines of DM and EM in Fig. 1, DM was found to be more appropriate than EM in epikarst spring. Conversely, both EM and DM had good fit in the EC, MC and soil baseflow. The MRTs calculated by EM were shorter than those calculated by DM. This resulted from that the MRT of soil water calculated by EM was invalid due to the existence of frequent water exchange in soils. Based on DM, EC and MC had similar MRTs of 71 and 70 weeks, respectively, and they were longer than those of soil baseflow (64 weeks) and epikarst spring (56 weeks). Moreover, soil baseflow had longer MRT than epikarst spring. The MRT of soil waters ranged between 3 to 60 weeks and increased with increase in soil depth except the SD-3. There were weathered sandy soil layers at depths of 60 and 80 cm in the SD-3, which made water movement more unusual than the other sites.

## Discussion

### Epikarst water movement

In karst regions, the epikarst acts as a temporary storage and distribution system for water infiltrating into karst systems^8^,^15^. The MRT had some negative relationship with size and quantity of conduits in aquifer^41^. In limestone or rock catchment, the MRT of spring water largely depended on the size and quantity of conduits and ranged commonly from few weeks to several months^5^,^19^,^32^,^37^. However, in our study area (dolomite), the MRT of spring calculated with DM was 56 weeks (more than one year), which was similar to the results reported by other studies^5^,^6^,^19^. The long MRT of water in the epikarst and EC indicates this dolomite karst aquifer had less conduits and fractures to allow fast flow^5^,^38^ than those in typical limestone karst aquifers. Micropores and fissures water, which was characterized by long MRTs^5^,^37^, tended to be the major part of epikarst water storage in dolomite aquifers. Moreover, water mixing was sufficient due to diffusion exchange between mobile and immobile zones. Perrin *et al.*^3^ found that if recharge exceeded a given threshold of epikarst, excess infiltrated water bypassed the soil and epikarst and reached the saturated zone as fresh flow with high CV and short MRT. The apparent threshold of epikarst in this study was high enough to prevent bypass flow and maintain slow flow. In addition, the quick and short response of outlet flow to rainfall commonly accompanied with the existence of conduits. However, such response was longer than anticipation. These supports our hypothesis that dolomitic epikarst would not have rapid hydrological processes, and rainwater infiltrated quickly into epikarst with bare or shallow soil cover but stayed for a long time rather than flowed out. The great numbers of pores and small fissures, which were characterized by slow flow, might be the reason^9^. The interaction among waters from different flows increased the MRT of fast water. Consequently, this type of epikarst has a large water storage capacity and contributes to delay the hydrological processes in the dolomitic karst groundwater system.

The MRT was influenced by multiple factors such as gradient, landscape organization, contributing area and flow-path length^32^,^33^,^34^. Similar to the argument reported by Soulsby *et al.*^34^ the contributing area is less influential than that previously considered. One of the reasons in this study was that the landscape organization controlled the MRT of catchment. Another reason was that the depression mixed water well due to the deep soil. Dewalle *et al.*^22^ and O’Driscoll *et al.*^2^ found that annual average hydraulic diffusivity for water movement in the soil had positive relationship with soil depth. Well mixing water caused even distribution of MRT in the depression.

### Model fitting

The fit of models reflected the water movement pattern of subsurface system to a certain degree^5^,^29^,^39^. The DM seemed to be much closer to the reality in the study area. This was because the EM assumed that there is no exchange of tracer between the flow lines. However, water movement in soils was difficult to satisfy this premise, unless the new water fraction contributed little on the output water resources^6^,^7^. Obviously, the soil in the slope was impossible, but soil in the depression was exception.

The low slope gradient, clay texture and deep soil thickness made the depression be a good water mixing reservoir. This was likely why the EM fit lines of creek, outlet and soil baseflow, which contained water from the depression, were better than that of the epikarst spring. However, this situation could not resolve the model error of EM for MRT of soil water. The slope epikarst water converged easily and dominated the water resources in the depression, because the recharge area and thickness of epikarst were larger than those of soils in the study area. These results were similar to the condition of no soil cover on the epikarst^15^,^19^. Consequently, the MRTs calculated by EM were shorter than those calculated by DM.

### The impact of soil in the depression

Similar to previous studies, the major recharge of the creek and shallow groundwater occurred during the rainy season^1^,^2^. This was because most of the rainfall came from the rainy season and karst slopes had fast infiltration rate and rare overland flow^13^,^39^. However, there was a difference of recharge portion among creek, soil baseflow and epikarst spring. Compared with the soil baseflow, dry-season rainfall occupied a smaller recharge portion in epikarst spring. This is attributed to the difference between the soil baseflow and epikarst spring in that the former contained more deep soil water from the depression. The soil zone in the depression either intercepted input rainwater from the rainy season or increased the input rainwater from dry season. The former is the more likely reason. On the one hand, high infiltration rates and rare overland flow on the karst slopes in this study area were found and implied that such shallow soils facilitated the recharge of underlying epikarst for an entire hydrological year^39^. On the other hand, relative to the dry season, rainfall was more frequent during the rainy season. This made excess-infiltration runoff more common during rainy season. The hydraulic conductivity value for sand is several times greater than that for clay loam, indicating that the depression was the major place to intercept excess infiltrated rainy-season rainfall^41^. Also, the slope position had important influence. The depression which had lower slope gradient than upslopes, tended to yield runoff much easier^41^,^42^.

Soils in the depression play an important role in water mixing due to the presence of capillary water storage^3^. Moreover, deep soil layer had higher water storage capacity than shallow soil layer because of fewer large apertures and more micropores^17^,^41^,^42^. This meant that the soil water storage had a positive relationship with soil thickness under the condition of similar soil texture. Soil in the depression decreased the recharge of underlying epikarst water and increased the overland flow at the yearly scale. In the combination of long MRT of the EC, it was found that the conduit water (fast water) was few. Consequently, the linear mixing model could be accepted in the study area, because rainwater mainly infiltrated into slow reservoir^43^. For example, Herrmann *et al.*^19^ and Zarei *et al.*^44^ also found that two-component hydrograph separation model got a good result of distinguishing old water and new water of the stream in karst basin.

The soil in the depression extended the MRT of subsurface water compared to upslope soil. This is because the topography and soil distribution in the depression was similar to riparian. Although there were no previous studies of this site, similarly free-draining riparian zone soils had an ability to dampen tracers more effectively^45^. Soulsby and Tetzlaff 4 also found that MRT was more closely correlated with riparian soil cover – even quite simply defined as that within 50 m of the river channel – than total cover of responsive soils. This relationship is consistent with the importance of the topography and soil distribution of catchment, particularly in relation to the connectivity between more responsive soils and the river channel network.

## Conclusions

The seasonal recharge and mean residence time (MRT) of water in the end of creek (EC), the middle of creek (MC), soil baseflow, epikarst spring and soils were revealed by their seasonal variations of deuterium contents. The major recharge of subsurface and surface water occurred during the rainy season, and recharge rate of rainy-season rainwater was higher than that of dry-season rainwater. Deep soil (>60 cm) in the depression had higher water-holding capacity than shallow soil at the footslopes. The creek was recharged not by direct rainfall but by rainfall through the epikarst zone. This reflected that karst catchment had high infiltration rate. The MRT of all water bodies calculated with DM was longer than one year. This indicated that epikarst had poor connectivity and good water-holding capacity, and it could maintain the continuous recharge to major surface water. Furthermore, this also reflected the key role of epikarst in water circulation in the karst catchment. Soil baseflow had longer MRT than epikarst spring. This demonstrated that deep soil in the depression made input water stay in subsurface system for longer time and intercepted part of the rainy-season rainfall. Comparatively speaking, the contributing area had little impact on MRT than soil thickness in the small karst catchment. The results of this paper should improve our understanding of karst hydrological processes at catchment scales.

## Materials and methods

### Site descriptions

**Figure 3:**
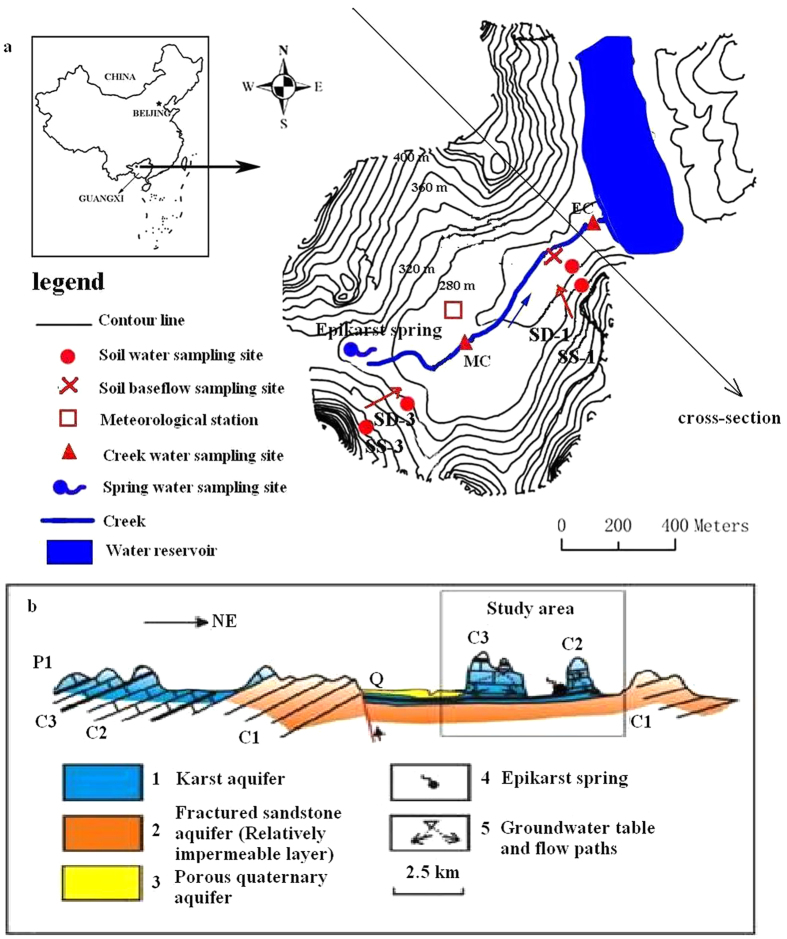
Schematic map of sampling sites (**a**) and vertical cross-section of the geohydrologic background (**b**) in small karst catchment of Huanjiang County of northwest Guangxi, China. Schematic map of sampling sites (**a**) was generated by software ArcGis and ArcView, and the positions of sampling sites were located by GPS. Geohydrologic background (**b**) was drawn by using plot software CAD based on data of field investigation.

The study was conducted at the Huanjiang Observation and Research Station for Karst Ecosystems under the Chinese Academy of Sciences (24°43′–24°44′N, 108°18′–108°19′E) in Huanjiang County of northwest Guangxi, southwest China (Fig. 3). The experimental site is a typical peak-cluster depression area covering 1.01 km^2^, and it is relatively closed area. About 60% of slope land has a slope gradient greater than 25° and the elevation ranges from 272 to 647 m above sea level. This region experiences a subtropical mountainous monsoon climate. The average annual rainfall is 1389.1 mm with 71% falling in the wet season from May to October. This period is defined as the rainy season. The average annual temperature is 18.5 °C. The soil depth in the depression and on the hillslope varies from 20 to 160 cm and from 0 to 50 cm, respectively. On most parts of the top hillslope, there is little soil cover but exposed bedrock. The shallow and discontinuous soils have been developed from dolomite and contain significant amounts of rock fragments. Soils are well-drained, gravelly and calcareous, and have a clay to clay loam texture (25–50% silt and 30–60% clay) with stable infiltration rate varying from 0.43 to 4.25 mm/min^39^. Soil organic matter content is relatively high ranging from 2.2% to 10.1%, and pH varies between 7.1 and 8.0. The cover of exposed bedrock range from 15% to 30%, and some rock outcrops covered mostly by deep-rooted trees are large (2–10 m in height).

The study area is located at a wide and gradual edge of syncline from northwest to southeast. The exposed stratum is gray dolomite. There are gray and black limestones and yellow sandstone underneath the dolomite. The gray dolomite is widely distributed in the area and characterized by good water-holding capacity. There is one continuous and two discontinuous epikarst springs locating at the bottom of the dolomite hillslope. A surface water reservoir lies on yellow sandstone in the northeast of the study area. The yellow sandstone is considered to be a relatively impermeable layer and water losses from the small catchment were limited. The groundwater table changed seasonally and was often below 1–3 m depth in the depression^46^.

All residents have moved away and the cultivated lands have been abandoned since 1985. The vegetation consists of grassland and sparse shrubland. However, there are patches of zonal dense scrub and forest lands with a high cover of exposed bedrock, especially in the southwestern parts. Overland flow is low and the corresponding runoff coefficient is often less than 5% on the hillslopes under various land use types.

### Water sampling

Rainfall and water samples from creek, soil baseflow and one epikarst spring (Fig. 3) were sequentially collected weekly within the study site from April 10, 2011 to April 9, 2013. The number of water samples from each water body was 99. In order to estimate the influence of contributing areas, two sampling sites were chosen in the creek. One located at the end of the creek (EC, outlet of the catchment), and one in the middle of the creek (MC). Soil baseflow was collected with a zero-tension PVC tube at the soil-bedrock interface (about 100-120 cm depth) near the EC in the depression. In order to avoid the impact of creek and depression, epikarst spring was chosen in the upper area of catchment. The precipitation sampling devices in the present study are similar to those used by IAEA and consist of a 150-mm-diameter funnel connected to a 0.5 L brown bottle^47^. An air outlet tube was welded at the lower part of the funnel. In order to avoid evaporation of water samples, paraffin was used at every joint. The weekly rainfall amount was obtained from a meteorological station of the study site. The water sampling sites are illustrated in Fig. 3. Weekly water samples were preserved at 4 °C to prevent evaporation.

Soil waters were sampled at 5 different depths from 20 to 100 cm in 4 sampling sites and collected weekly between April 10, 2011 and February 28, 2013. Two sites (SS-1 and SS-3) located in the slopes and the others (SD-1 and SD-3) in the depression. They formed two sampling lines, and one (SS-3 and SD-3) was near the epikarst spring (Fig. 3). This design was good for evaluating impact of slope position on soil water movement. Soil water was sampled by using tension lysimeters (produced by Institute of Geographic Sciences and Nature Resources Research, CAS) which consist of suction lysimeters, ceramic porous cup, sampling bottle, tubes and pressure monitor^48^. A small hand pump was connected to the device and about an 80-Kpa vacuum was provided within the bottle.

In order to confirm high rainfall is more easily intercepted by soils in the depression than those on slopes and the corresponding surface runoff varies, the rainfall-runoff responses of the EC and epikarst spring were recorded hourly after two different typical rainfall events in 2012: April 5 (low rainfall) and March 29 (high rainfall). The discharges of the EC and epikarst spring were measured by PS 1000 automatic water level monitoring probe (Greenspan) and they were transformed to discharge by weir.

### Deuterium and oxygen-18 analysis

Deuterium and oxygen-18 values of water samples were analyzed by the DLT-100 liquid water isotope analyzer (Los Gatos Research (LGR), Inc., model 908-0008), a liquid water isotope analyzer, at the Key Laboratory of Agro-ecological Processes in Subtropical Regions belonged to the Institute of Subtropical Agriculture under the Chinese Academy of Science. Results were reported in δ notation relative to V-SMOW as


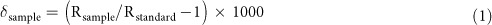


where R_sample_ and R_standard_ are the ratio D/H or O^18^/O^16^ of measured sample and standard sample, respectively. The standard deviation for repeated measurements was ±1‰ for δD and ±0.2‰ for δ^18^O.

### Water recharge portion analysis

Water recharge portion is defined as recharge contribution of dry-season rainwater to different water bodies on year scale. It is calculated by two end-member model^52^. On a year scale, rainwaters from dry and rainy seasons are identified as two unique original water resources to surface and subsurface water in the study area. The relationship between input and output can be represented by as


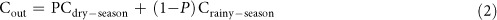


where C_out_, C_dry-season_ and C_rainy-season_ are *δ*D or δ^18^O value of water bodies, dry-season rainfall and rainy-season rainfall, respectively. P is recharge portion for year scale. The C_out_ is average weighted values with discharge during the stable discharge period. Because discharge of epikarst spring varies little under the baseflow condition, the C_out_ of epikarst spring is equal to unweighted value. C_dry-season_ and C_rainy-season_ are average weighted values with rainfall amount during dry and rainy seasons, respectively.

Additionally, the two end-member model also can be used to calculate the percentage of new water in storm-event. The relationship between input and output can be represented by as


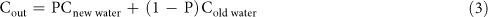


where C_out_, C_new water_ and C_old water_ are weighted *δ*D or δ^18^O value of output water, new water and old water, respectively. P is percentage of new water. The weighted *δ*D or δ^18^O value of new water and old water is replaced by that of rainfall and baseflow water, respectively.

### Mean water residence time analysis

Two lumped parameter mathematical models, i.e., DM and EM (version 3.1), were used to determine MRT of soil water at different depths and positions based on long-term isotope data^27^. A functional relationship between input and output can be represented as


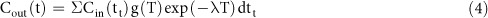


where C_out_ and C_in_ are weighted *δ*D and *δ*^18^O values of different water bodies and rainfall, respectively; g(T) is system response function, which specifies residence time distribution of water within the system; t_t_ is the time of entry; and T = t-t_t_ is the MRT of water, which can be calculated from system response function through calibrating models. Based on sufficient data of C_out_ and C_in_, the g(T) can be calculated.

In the DM, the following uni-dimensional solution in the flux mode to the dispersion equation for a semi-infinite medium was used as the response function


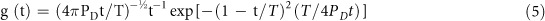


where P_D_ is the apparent dispersion parameter, which mainly depends on the distribution of travel times. The higher values reflect more inhomogeneity and greater width of transit time distributions. Consequently, the T can be obtained finally.

In the EM approximation, the flow lines are assumed to have the exponential distribution of transit times, i.e. the shortest line has the theoretical transit time close to zero, and the longest line has the transit time close to infinity. It is assumed that there is no exchange of tracer between the flow lines, and then the following response function is obtained:


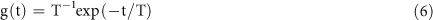


The accuracy of fit of simulations to the experimental data was computed using SIGMA function


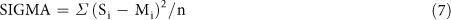


where S_i_ and M_i_ are simulated isotope values and measured isotope values, respectively; and n is the number of samples. The lower SIGMA value means a better fit.

In humid area, where evaporation is weak, the deuterium and oxygen-18 contents have strong linear correlations^19^,^27^. The isotopic enrichment due to fractionation processes is little during the evaporation. The results obtained from oxygen-18 and deuterium values met this situation and they were similar in our study area. However, in karst regions, Meißner *et al.*^50^ found that oxygen-18 content is greatly affected by the soil carbonate while deuterium content is not. Obviously, this uncertainty needs to be avoided in our study area. Indeed, many previous studies only chose deuterium as stable isotope^27^,^50^,^51^. Consequently, the results obtained by the analyses of deuterium contents were introduced in this paper.

### Soil depth survey

In order to get a better understanding of the role soil cover has in water circulation in the catchment and the interaction between soil water and epikarst water, the distribution of soil depth was investigated. In total, there were 151 soil plots, each with a dimension of 80 m × 80 m. For the purpose of equal distribution of soil plots in the study area, soil plots were designed by ArcGis. Soil depths were obtained by using surveying rod.

## Author Contributions

H.K. and C.-H.S. designed the study, collected and analyzed the data, and wrote the manuscript. N.-Y.P. and W.-K.L. discussed the experimental design and manuscript writing.

## Additional Information

**How to cite this article**: Hu, K. *et al.* Seasonal recharge and mean residence times of soil and epikarst water in a small karst catchment of southwest China. *Sci. Rep.* **5**, 10215; doi: 10.1038/srep10215 (2015).

## Figures and Tables

**Table 1:** The distribution of the different weekly amount of rainfall between rainy and dry seasons from April, 2011 to April, 2013.

**Rainfall**	**0-10 mm (Weeks)**	**10-20 mm (Weeks)**	**20-30 mm (Weeks)**	**30-40 mm (Weeks)**	**40-50 mm (Weeks)**	**50-100 mm (Weeks)**	**>100 mm (Weeks)**	**Total (Weeks)**
Dry season	22	18	3	6	3	2	0	54
Rainy season	21	4	6	4	4	9	6	54

**Table 2:** The distribution of soil depth in different slope positions in the study area.

**Slope position**	**Number of plots**	**Average soil depth (cm)**	**Standard deviation (cm)**
Upslope	38	20.8	9.8
Midslope	38	25.2	10.9
Downslope	38	36.9	20.0
Depression	37	63.8	35.0

**Table 3:** The average deuterium content, CV and recharge portion of entire year during dry-season in rainfall and different water bodies from April 10, 2011 to April 9, 2013.

**Samples**	**Depth (cm)**	***δ*****D (‰, SMOW)**	**CV (%)**	***δ*****D**_**dry**_ **(‰, SMOW)**	***δ*****D**_**rainy**_ **(‰, SMOW)**	**Portion (%)**
Rainfall	—	−31.65	96.79	−11.39	−50.91	29.53
EC		−42.34	10.59	−42.91	−42.01	21.69
MC		−42.49	12.32	−42.87	−42.48	21.31
Soil baseflow	—	−42.03	9.97	−41.41	−42.29	22.47
Epikarst spring	—	−44.11	8.43	−45.20	−43.22	17.21
SS-1	20	−30.79	60.19			50.91
	40	−37.25	40.64			34.56
	60	−45.98	26.73			12.47
	80	−46.56	28.97			11.01
	100	−46.79	27.5			10.42
SS-3	20	−26.59	91.22			61.54
	40	−34.17	51.17			42.36
	60	−46.48	26.92			11.21
	80	−47.76	21.15			7.97
	100	−46.99	19.48			9.92
SD-1	20	−25.82	69.19			63.49
	40	−34.87	23.61			40.59
	60	−40.98	15.82			25.13
	80	−43.89	8.07			17.76
	100	−40.56	8.74			26.19
SD-3*	20	−29.55	55.27			54.05
	40	−33.56	43.34			43.90
	60	−44.31	35.23			16.70
	80	−43.24	31.54			19.41

*δ*D, *δ*D_dry_ and *δ*D_rainy_ meant average values of annual, dry-season and rainy-season.

^*^meant weathered sandy soil layer at 60-80 cm depths in the SD-3.

**Table 4:** The MRT of different water bodies calculated by DM and EM, respectively from April 10, 2011 to April 9, 2013.

		**DM**			**EM**	
**Samples**	**Depth (cm)**	**T (weeks)**	**P**_**D**_	**SIGMA**	**T (weeks)**	**SIGMA**
EC	—	71	0.06	0.4701	45	0.3919
MC		70	0.05	0.6431	41	0.5363
Soil baseflow	—	64	0.05	0.4093	47	0.4131
Epikarst spring	—	56	0.09	0.4550	41	0.5599
SS-1	20	10	2.00	0.8454		
	40	10	1.50	1.7860		
	60	24	0.03	2.7765		
	80	28	0.01	3.0080		
	100	31	0.02	2.3959		
SS-3	20	3	1.35	1.1120		
	40	9	2.40	1.7678		
	60	9	0.01	3.5505		
	80	9	0.04	3.6722		
	100	12	0.01	4.1931		
SD-1	20	6	1.20	1.3770		
	40	18	0.50	1.2942		
	60	57	0.12	1.0327		
	80	58	0.08	0.9067		
	100	60	0.11	0.6451		
SD-3*	20	10	2.50	1.1958		
	40	11	2.50	1.9297		
	60	10	0.01	4.3131		
	80	9	0.01	3.9500		

^*^meant weathered sandy soil layer at 60-80 cm depths in the SD-3.
